# Supplementary material for: Exploring the relationship between pancreatic fat and insulin secretion in overweight or obese women without type 2 diabetes mellitus: A preliminary investigation of the TOFI_Asia cohort
Source: PLoS One. 2022 Dec 30;17(12):e0279085. doi: 10.1371/journal.pone.0279085 (PMC9803309; doi:10.1371/journal.pone.0279085)
Supplement: Attachment — Submitted filename: Response to Reviewer Comments.pdf [file pone.0279085.s002.pdf]

Reviewers' comments:

We would like to express our gratitude to the reviewer for providing us with insightful comments, and their efforts towards improving our manuscript. We have addressed and provided detailed responses to the feedback received below in the highlighted clean and track change versions of the revised manuscript. Please note that all pagination and line numbers refer to the submitted clean highlighted revised version.

Comments to the Author

In this study, the authors aimed to determine whether pancreatic fat negatively affects beta cell function in women with overweight or obesity but without T2D. Twenty women with NGT or IFG were included in the study. Low and high pancreatic fat was assessed by MRI and insulin secretion was assessed by ivGTT. Use of ivGTT and MRI to assess insulin secretion and pancreas fat is an advantage of the study. However, there are several major critiques.

1. The study design was not clearly described. If this is a subpopulation analysis of the original study (TOFI Asia cross-sectional study), brief description of the original study should be included in the methods section. The sample size calculation should also be described clearly.

AUTHOR RESPONSE: We note the comment by the reviewer and agree that this information was not clearly presented. We have now briefly expanded on this in the **MATERIALS AND METHODS Study design and protocol** section on **Page 9, Line 156–164** "*All women enrolled in the TOFI_Asia MR cohort attended the HNU clinic following an overnight fast; body weight, height, waist and hip circumferences, and blood pressure (BP) were recorded, and a fasted blood sample for determination of* HbA_{1c} *collected, stored at -80°C pending batch analysis on completion of the study using capillary electrophoresis (Cap2FP, IDF, France). Total and abdominal adiposity were assessed using dual-energy X-ray absorptiometry (DXA) (iDXA, GE Healthcare, WI, USA) at the Body Composition Laboratory, University of Auckland. Detailed abdominal MRI/S scans were conducted in the fasted state, within 1 week of the clinic visit, on a 3T Magnetom Skyra scanner, VE 11A (Siemens, BY, Germany) at the Centre for Advanced MRI (CAMRI), University of Auckland..*"

And further on **Page 11, Line 205-222** "*Following MR scanning, women who provided additional written informed consent were scheduled for their ivGTT within 7 days. A stepped insulin secretion test was performed as previously described (14, 33). In fasted participants, two 18G venous cannulae were inserted into the antecubital vein for infusion and the contralateral wrist vein for arterialised blood sampling. The hand was kept warm during the entire test to maintain arterialisation of venous blood. Two consecutive, 30-min square-wave steps of hyperglycaemia (2.8 and 5.6 mmol/L above baseline) were achieved using a bolus dose of 25% dextrose (Baxter Healthcare, NSW, Australia). The priming bolus dose for each step was calculated as 0.378 g/kg body weight of 25% dextrose and administered over one min to avoid thrombophlebitis. Post-administration of the bolus dose, plasma glucose concentrations were maintained at the desired plateau with a variable 25% dextrose infusion (Carefusion,*

Alaris®, BBRK, UK). Blood samples for determination of plasma glucose, insulin and C-peptide were obtained every 2 min for the first 10 min and every 5 min over the next 20 min of each step. Two Caucasian women did not successfully complete the second 30-min of the test, due to the inability to obtain arterialised venous blood. We were unable to obtain sufficient blood samples for analysis and estimation of insulin secretion rate (ISR) from one Caucasian woman, who was therefore excluded from the statistical analysis. Therefore, final data presented are for 19 women (9 Chinese and 10 Caucasian) (Fig. 1)."

Whilst there was no a priori sample size calculation conducted for the study, we performed an informal assessment of sample size based on a similar prior study published by members of our research team which also investigated beta cell function in a group of 11 individuals: **Lim, Hollingsworth, Aribisala, et al., Reversal of type 2 diabetes: normalisation of beta cell function in association with decreased pancreas and liver triacylglycerol. Diabetologia 2011, doi: 10.1007/s00125-011-2204-7.** The informal sample size assessment was based on first-phase insulin response at baseline (mean: 0.19 ± 0.02 SEM nmol min^{-1} m^{-2}) following dietary intervention (mean: 0.46 ± 0.07 SEM nmol min^{-1} m^{-2}). Based on the Lim data, to detect effect size 0.27 nmol min^{-1} m^{-2} as significant would require a minimum of n = 10 individuals. Modelling based on a more conservative effect size of ~50% of the Lim data (0.14 nmol min^{-1} m^{-2}) and variance at pre-intervention baseline (0.07 nmol min^{-1} m^{-2}) generated a required sample size of n = 14 for 2 independent groups, and effect size of ~25% of Lim generated a required sample size of n = 23 per group. Based on these estimates we continued to recruit individuals from the TOFI_Asia cohort until we had enrolled 22 individuals.
Hence, all women from the TOFI_Asia MR study who underwent an MRI scan and provided informed consent to undertake a 1 hr-ivGTT were included in the cohort. We have now clarified this in the **MATERIALS AND METHODS Study Population** section on **Page 8, line 140 – 153** "*Of the 202 women who were enrolled in the TOFI_Asia cross-sectional study, a subset of 68 women (34 Chinese and 34 Caucasian) successfully joined the TOFI_Asia MR cohort and underwent abdominal MRI scans for assessment of pancreatic fat. Of these, 22 women (9 Chinese and 13 Caucasian) provided written informed consent and were enrolled into the current ivGTT cohort, for determination of pancreatic β-cell function as a measurement of insulin secretion (Fig. 1). Informal power calculations showed a minimum of 10 individuals would be required to detect a physiologically significant increase in first phase insulin response. Inclusion criteria was based on the TOFI_Asia MR study (26) and as such the ivGTT cohort were 20-70 years of age, body mass index (BMI) 20–35 kg/m^2, normoglycaemic (fasting plasma glucose < 5.6 mmol/L) or impaired fasting glucose (IFG; 5.6 – 6.9 mmol/L) (27) and MRI-assessed percentage pancreatic fat (PPF) classified as < 4.5 % or ≥ 4.5 %. PPF could not be analysed from scans obtained from 2 women due to motion related artefact, and hence 20 women (9 Chinese and 11 Caucasian) had an assessment of pancreatic β-cell function using ivGTT and were included in the current analyses (Fig. 1).*"

2. The small sample size is a major limitation. In addition, the inclusion of different glucose tolerance status (NGT and IFG) and different ethnic groups made interpretation of data more complex. It is difficult to conclude based on this small sample size and the authors are highly recommended to increase the sample size to lead more solid conclusion. At least, the term "preliminary results" or "pilot study" should be included in the title.

AUTHOR RESPONSE: We note the comment made by the reviewer and are in agreement that sample size is indeed a limitation of the study and have acknowledged this on **Page 18,**

Line 385-386 "*Limitations of this study include the small, single gender cohort which may not allow generalization of the data and findings*." We have taken on board the reviewers suggestion and have now revised the title of the paper on **Page 1, Line 1-3** "*Exploring the relationship between pancreatic fat and insulin secretion in overweight or obese women without type 2 diabetes: a preliminary investigation of the TOFI_Asia cohort*"

We also note the reviewer's suggestion to increase the sample size. Our study was completed in 2017 and although presently unable to add to the present cohort, we are undertaking 3-yr follow-up assessments on the cohort which we are looking to publish in early 2023.

Additionally, although we have a small sample size of 20 women (9 Chinese and 13 Caucasian), a strength of our study is that we undertook statistical analyses specifically using repeated measures linear mixed models, that were adjusted for ethnicity and the average fasted glucose concentration (reflective of endogenous glucose production), in women identified with PPF above and below our defined high/low PPF cut-off. This has been detailed in the **Statistical Analysis** section **Page 12, line 242-246**, "*Changes from baseline (Δ) in glucose, insulin, C-peptide and ISR over the 60-min test were evaluated using repeated measures linear mixed models, adjusted for ethnicity and the average baseline (-10 min and 0 min) concentration, in women identified with PPF above and below our defined high/low PPF cut-off. The interaction effect between the two PPF groups and time points was tested in the mode*l."

3. As the incremental AUCs for both first and second phase ISR were not significantly different between the high and low PPF groups, their conclusion "early signs of decreased biphasic insulin secretion in those with pancreatic fat >=4.5% may be of clinical importance" was not supported by the data and should be removed.

AUTHOR RESPONSE: We have taken on board the reviewer's feedback and agree that the conclusion unsupported and requires to be revised in the Conclusion of the Abstract. The sentence has been re-phrased on **Page 4, Line 64-67** "*In women with overweight or obesity but without type 2 diabetes, PPF did not modify β-cell function as determined by ivGTT-assessed ISR. However, the salient feature in biphasic insulin secretion in those with ≥4.5% PPF may be of clinical importance, particularly in early stages of dysglycaemia may warrant further investigation.*"

4. Uploaded figures are blur and difficult to see. More clear images should be provided.

AUTHOR RESPONSE: We thank the reviewer for the attention to detail. We would like to intimate the reviewer that upon receipt of the reviewer comments we were informed by the Staff Editor (Hanna Landenmark) that the blurriness in the reviewer PDF was an artefact of the PDF generation. They confirmed that the original images that we uploaded onto the PLOS One system are of high enough quality for the Journal and hence informed us that we did not need to revise the images further.
